# Investigating acoustic numerosity illusions in professional musicians

**DOI:** 10.3758/s13423-024-02496-2

**Published:** 2024-04-10

**Authors:** Alessandra Pecunioso, Andrea Spoto, Christian Agrillo

**Affiliations:** 1 Department of General Psychology, University of Padova, Via Venezia 8, 35131 Padova, Italy; 2 Padua Neuroscience Center, Padova, Italy

**Keywords:** Solitaire illusions, Psychology of music, Gestalt, Perception, Numerical estimation

## Abstract

Various studies have reported an association between musical expertise and enhanced visuospatial and mathematical abilities. A recent work tested the susceptibility of musicians and nonmusicians to the Solitaire numerosity illusion finding that also perceptual biases underlying numerical estimation are influenced by long-term music training. However, the potential link between musical expertise and different perceptual mechanisms of quantitative estimation may be either limited to the visual modality or universal (i.e., modality independent). We addressed this question by developing an acoustic version of the Solitaire illusion. Professional musicians and nonmusicians listened to audio file recordings of piano and trombone notes and were required to estimate the number of piano notes. The stimuli were arranged to form test trials, with piano and trombone notes arranged in a way to form the Solitaire pattern, and control trials, with randomly located notes to assess their quantitative abilities in the acoustic modality. In the control trials, musicians were more accurate in numerical estimation than nonmusicians. In the presence of illusory patterns, nonmusicians differed from musicians in the esteem of regularly arranged vs. randomly arranged notes. This suggests that the association between long-term musical training and different perceptual mechanisms underlying numerical estimation may not be confined to the visual modality. However, neither musicians nor nonmusicians seemed to be susceptible to the acoustic version of the Solitaire illusion, suggesting that the emergence of this illusion may be stimulus and task-dependent.

**Supplementary Information:**

The online version contains supplementary material available at 10.3758/s13423-024-02496-2.

## Introduction

The term ‘numerosity illusions’ refers to a category of visual illusions characterized by a misperception of items presented in the scene. This type of illusions is considered a powerful tool to investigate the perceptual biases underlying numerical estimation of human (Bertamini et al., 2023; Ginsburg, 1980) and nonhuman animals (Beran, 2006; Lõoke et al., 2020). The Solitaire illusion, originally studied by Frith and Frith (1972), represents the most investigated numerosity illusion (e.g., Agrillo et al., 2014, 2016; Parrish et al., 2016, 2019). This occurs when observers misperceive the relative number of two different colours of otherwise identical objects in intermingled sets. Items forming a single cluster are supposed to be overestimated. In the most traditional version of the Solitaire array, one set of dots is centrally located in the visual scene and forms a single cluster because of proximity and good continuation, compared with the other set of items forming small separate clusters. The illusion has been reproduced in the auditory modality too (Prpic & Luccio, 2016), raising the intriguing possibility that the above-mentioned Gestalt principles may determine a misperception of numerosity also in auditory modality. This result, together with other perceptual effects reported in the visual and auditory modality (e.g., Gestalt principles of proximity and good continuation in the scale illusion; Deutsch, 1974) and good continuation in the illusory continuity tones (Riecke et al., 2008), suggests that perceptual mechanisms may be universal and not modality dependent. In the case of numerical estimation, however, there is a debate as to whether numerical acuity variates (Tokita et al., 2013; vanMarle & Wynn, 2009) or not (Barth et al., 2003; Izard et al., 2009) based on the perceptual modality. According to some authors (Barth et al., 2003; Izard et al., 2009), the numerical acuity is domain independent; thus, no differences should be observed in visual and nonvisual modalities. On the other hand, Tokita et al. (2013) reported a different performance in numerosity judgments when stimuli were presented in the visual or auditory condition, advancing the hypotheses of multiple core number systems where visual and auditory numerosities would be mentally represented with different signal variabilities.

Perceptual mechanisms are known to be partially influenced by experience (Lu & Dosher, 2022). For instance, various studies have reported statistically significant, although small, associations between musical expertise and a better performance in visuo-spatial tasks (e.g., Raven’s matrices: Forgeard et al., 2008; subtest of the WISC-II: Rauscher et al., 1997). Musicians appear to outperform nonmusicians also in numerical tasks (e.g., nonsymbolic numerical estimation; Agrillo & Piffer, 2012). Musicians’ neural correlates during visuo-spatial and numerical tasks were found to be different from those of nonmusicians (Magne et al., 2006; Schmithorst & Holland, 2004; Sluming et al., 2007). Taken together, these results support the idea that, although the causal mechanisms are not known, music training may be associated with changes in neural circuits not primarily involved in music per se.

Pecunioso and Agrillo (2021) raised the question of whether long-term music training may also influence the perceptual mechanisms underlying numerical estimation. To address this issue, the authors investigated the susceptibility to the Solitaire illusion in musicians and nonmusicians by asking participants to estimate the number of blue dots in an array comprising yellow and blue dots. The dots were either randomly arranged (control trials) or located in a way to form the Solitaire illusion (test trials). In line with previous literature, Pecunioso and Agrillo (2021) found that participants overestimated the number of blue dots when they formed a single cluster. However, nonmusicians had a greater tendency than musicians to overestimate the numerosity of items forming a single cluster. This finding might be interpreted in the light of professional musicians exhibiting a perceptual advantage in numerical estimation and thus being less susceptible to the Solitaire illusion. In addition, the comparison between control (randomly arranged items) and test trials (orderly arranged items) raised the intriguing possibility that regular-random arrays might be another perceptual cue (beyond the formation of a single cluster) that impacts numerical estimation differently in musicians and nonmusicians. The effect of ordered arrays in numerical estimation is known as the Regular–Random Numerosity Illusion (RRNI), in which observers are inclined to overestimate ordered objects compared with those arranged randomly (Beran, 2006; Ginsburg, 1980). Results from Pecunioso and Agrillo (2021) indeed suggested that musicians appeared to be less fooled by the regular–random effect.

The potential link between musical expertise and different perceptual mechanisms of quantitative estimation may be either confined to the visuo-spatial domain or, instead, occur also in other perceptual modalities. To help shed light on this debate, in the current study, we present an acoustic version of the Solitaire illusion to musicians and nonmusicians. Originally developed by Prpic and Luccio (2016), the acoustic Solitaire illusion is characterized by audio files that reproduce an arrangement in pitch space similar to the linear arrangement of dots in the visual Solitaire illusion (Frith & Frith, 1972). The pitch space is defined by the relative pitch of two tones from two different musical instruments (i.e., piano and drums). The two types of notes corresponded to the different-coloured dots of the visual illusion. Prpic and Luccio (2016) provided the first evidence of a numerosity misperception with the auditory pattern. However, as participants were involved in relative numerosity judgments (more piano or drum sounds?), it was not possible to assess the exact magnitude of numerosity misperception.

Starting from the illusory stimuli developed by Prpic and Luccio (2016), we generated the auditory version of the Solitaire illusion with piano and trombone tones. Should musicians perform better than nonmusicians in the numerical auditory estimation, this would support the idea that musical training has a universal and modality-independent influence on numerical estimation. On the contrary, if we did not observe a difference between musicians and nonmusicians in the auditory modality, we might conclude that the perceptual mechanisms that permit musicians to be less susceptible to the Solitaire illusion (Pecunioso & Agrillo, 2021) are modality dependent and probably confined to the visual modality.

Lastly, we analysed the performance of musicians and nonmusicians with regular (the illusory patterns) versus random patterns of notes (control trials) to see whether the accuracy of the two groups differ when the target notes were regularly and randomly arranged. As far as we are aware, no study has currently investigated the equivalent acoustic version of the RRNI.

## Method

### Participants

Forty volunteers participated in the experiment and were assigned to a group of musicians or nonmusicians. Musicians (*N* = 20, eight males, between 18 and 50 years old, *M*_age_ = 25.05 years) were sampled at the Conservatory of Padua and Brescia (Italy). As in the study by Pecunioso and Agrillo (2021), they were performing artists who had graduated from the Conservatory (bachelor, master’s, or older diploma) and played a musical instrument for at least 10 years (Table 1). Nonmusicians (*N* = 20, nine males, between 21 and 26 years old, *M*_age_ = 23.40 years) were sampled and tested in Padua and Brescia. They had at least a high school diploma and declared they had neither received music education outside secondary school nor sang or played any instrument (see supplementary material for participants’ information). All participants declared to have normal hearing. In accordance with the Declaration of Helsinki, participants signed the informed consent before starting the experiment. The study was approved by the ethical committee of the University of Padova (protocol number: 2576).

**Table 1:** Musicians involved in the study: instrument played and level of expertise (onset and years of training)

Subjects	Instrument	Onset age of training	Years of consecutive training
1	Transverse flute	7	15
2	Violin	10	13
3	Transverse flute	12	14
4	Harp	5	21
5	Cello	4	17
6	Transverse flute	6	18
7	Oboe	11	13
8	Voice	7	15
9	Guitar	8	14
10	Cello	11	15
11	Voice	20	30
12	Violin	6	16
13	Bassoon	5	13
14	Violin	5	20
15	Piano	6	22
16	Saxophone	10	13
17	Trumpet	7	17
18	Violin	6	22
19	Piano	8	17
20	Guitar	7	15

### Acoustic Solitaire illusion experiment

#### Apparatus and stimuli

The testing setup included a personal laptop (ASUS K52J) and noise-cancelling headphones (Bose QuiteComfort wireless 35). The experiment was built and run with E-Prime 2.0. The task was conducted in quiet rooms located at either the Conservatory of Padua or at the home of participants in Brescia. Testing participants in different places was not meant to affect their performance as all subjects were placed in a quiet room and wore noise-cancelling headphones (used also to present the stimuli) and an eye mask as a strategy to limit visual inputs coming from the different environments. This latter strategy also allowed participants to focus on acoustic stimuli.

The stimuli consisted of a sequence of trombone and piano notes (.wav format, 75 dB SPL) created using MuseScore3 software (Version 3.0.1.20439). We chose piano and trombone tones because of their highly different timbre. Stimuli were made by three different notes: A (830.61 Hz), B (233.08 Hz), and E notes (77.82 Hz). In nonsymbolic numerical tasks, stimuli are commonly presented for a very limited amount of time to avoid the use of verbal counting (Agrillo et al., 2013; Halberda et al., 2008; Vetter et al., 2008). Therefore, unlike Prpic and Luccio (2016), whose tempo for each audio file was fixed at 120 bpm, we opted for a faster tempo of 200 bpm. Notes were arranged to form three different patterns (Patterns A, B, and C). These corresponded to spatial arrangements that compose the linear version of the Solitaire illusion (Frith & Frith, 1972, Fig. 1).

**Fig. 1:**
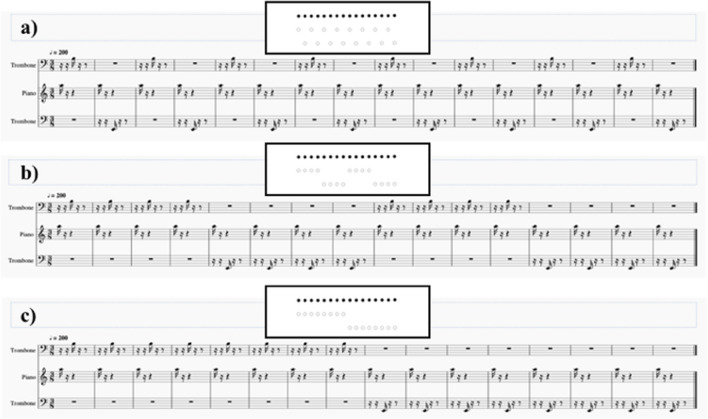
The three patterns (**a**, **b**, **c**) of the visual version of the Solitaire illusion (white and black dots) and the corresponding acoustic version (musical score)

Firstly, participants received a training phase in which six trials (2 for Pattern A, 2 for Pattern B‚ and 2 for Pattern C) were presented. These were composed of a total of 32 notes randomly located. Half of the trials included 10 piano notes and 22 trombone notes, whereas the other half presented the opposite arrangement (22 piano notes and 10 trombone notes). Two participants in the nonmusician group were excluded from the experiment at this stage, as they did not prove to understand the task. In the experimental phase, 144 trials were randomly presented. Stimuli were divided into control and test (illusory) trials. They again included 32 overall notes for each file. Control trials consisted of sequences of 14, 16, or 18 piano notes randomly arranged within Patterns A, B‚ and C (for a total of 108 control trials). Trombone notes were 18, 16‚ and 14, respectively. Test stimuli (16 piano and 16 trombone notes; piano and trombone notes were orderly presented) appeared 36 times, 25% of the total amount of trials. The limited prevalence of test trials aimed at preventing participants from understanding the regularity of test trials and in using nongenuine strategies of numerical estimation (e.g., by using the metre of the music score). Like the control trials, the test trials were arranged within the three patterns (Fig. 1) but were characterized (a) by the same number of piano and trombone notes and (b) specific orders of notes to form three acoustic versions of the Solitaire illusion investigated by Prpic and Luccio (2016). Pitch proximity is one of the strongest Gestalt mechanisms for clustering notes (Deutsch, 2013). Accordingly, in half of the test trials (*N* = 18), the piano notes had the same pitch in order to form a single cluster; in the other half, they formed two (smaller) clusters as two pitches were presented (see Table 2 for a summary of the types of stimuli and supplementary material for some examples of the auditory stimuli).

**Table 2:**
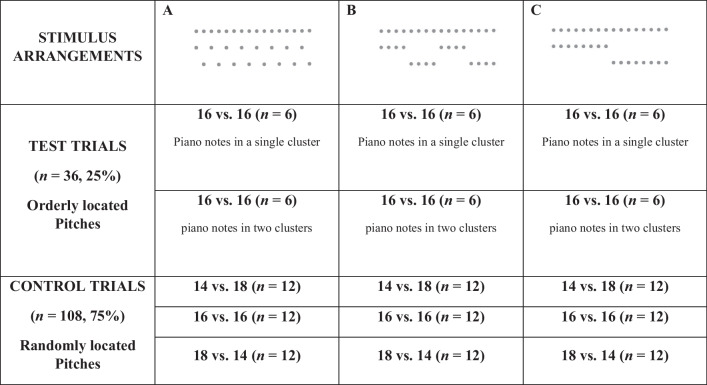
Summary of the stimuli presented for the three patterns (A, B, and C)

#### Procedure

To familiarize participants with the difference between the two timbres, a sequence of nine piano notes and a sequence of nine trombone notes, quarter notes (C) with a tempo of 60 bpm, were initially presented. The experimenter clearly explained to each participant which sequence was made by piano notes and which was made by trombone notes. Then, participants underwent a training phase consisting of six acoustic stimuli. After each sequence, participants were asked to estimate, as quickly as possible, how many piano notes they heard. The experimenter recorded the participants’ response. The following stimulus was played after the participant pressed the spacebar. Once the training phase was concluded, the experiment started. The experimental phase was divided into two blocks with 72 trials each. Participants were free to take a break after the first block, and, as soon as ready, they pressed the space bar to begin the second block of trials.

### Control experiment: Digit span test

The digit span test (a subtest of the Wechsler Memory Scale [WMS]) was performed to examine whether any potential difference between the two groups in the main experiment could be ascribed to differences in working memory and attention to the musical training. Specifically, we used the digit span forward (FS) and the digit span backward (BS) tasks: After the experimenter read a sequence of numbers, participants recalled the numbers forward (i.e., in the same order as presented) or backward (i.e., repeated in reverse order). Then, we recorded the overall span forward (FS) and backward (SB), as well as the total number of digits correctly remembered in the hardest trials, both forward (i.e., subscore of the digit span forward [SSF]) and backward (i.e., span subscore of the digit span backward [SSB]).

### Statistical analysis

#### Acoustic Solitaire illusion experiment

For the control trials, we computed the mean number of estimated piano notes for each stimulus type (from now on *absolute response*). Additionally, to understand the extent to which participant estimations deviated from the actual number of notes presented (i.e., the magnitude of numerosity misperception), the error rate of each participant’s response was calculated using the following formula [(participant’s response – target number)/target number] × 100 (Dormal et al., 2018; Pecunioso et al., 2020; Pecunioso & Agrillo, 2021). Thus, error rates are expressed as percentages of deviation from the target number with positive values representing an overestimation and negative values indicating an underestimation of the target number of notes. The error rates obtained were then averaged among the 12 trials of each type of stimulus (from now on *mean error rate*). A mean error rate equal to zero meant a correct estimation (for instance, error = −1 in 6 trials and error = 1 in the other 6 trials led to a mean error rate equal to 0). Also, in the test trials, we obtained participants’ mean absolute responses and mean error rates for each stimulus type.

We analyzed data by means of JASP 0.16.1 (JASP Team, 2022). For the control trials, to test whether nonmusicians and musicians had different abilities in estimating the number of notes in the different patterns, we estimated a repeated-measures analysis of variance (ANOVA), 2 (group: nonmusicians, musicians) × 3 (target number of notes to be estimated: 14, 16, 18) × 3 (pattern: A, B, C) for both the mean error rate and absolute response. Furthermore, we conducted planned orthogonal contrasts to test the effect of the group in the number of estimated notes when variating the target number. We expected that nonmusicians and musicians systematically and significantly differed in the estimations irrespective of the target number.

In the illusory trials, we estimated a repeated-measures ANOVA, 2 (group: nonmusicians/musicians) × 3 (arrangement: one cluster of 16 piano notes/two clusters of 8 piano notes each/16 piano notes, randomly arranged taken by the control trials) × 3 (pattern: A, B, C) for both mean error rates and absolute responses. We conducted planned orthogonal contrasts to test the effect of the group in the number of estimated notes when variating their position with always the same number of items (16).

All results were considered significant with *p* < .05. Moreover, we used the η^2^ and the Vovk–Sellke maximum *p* ratio (VS-MPR) for evaluating the maximum possible odds in favour of H_1_ over H_0_ as measures of the size of the effect.

#### Control experiment: Digit span test

To assess whether musicians and nonmusicians differed with respect to digit span forward and backward, we ran independent *t* tests for FS, BS, SSF, and SSB.

## Results

### Acoustic Solitaire illusion experiment

#### Control trials

A significant main effect of the group for the mean error rate, *F*(1, 36) = 5.835, *p* = .021, η^2^ = .081, *VS-MPR* = 4.548, was observed, indicating that, overall, nonmusicians underestimated piano notes more than musicians (nonmusicians: *M* = −11.952, *SD* = 16.942; musicians: *M* = −2.942, *SD* = 13.578). Moreover, participants’ mean error rate significantly differed across various levels of the target number, *F*(2, 72) = 989.076, *p* < .001 , η^2^ = .363, *VS-MPR* = 6.996e^49^. This result must be interpreted considering the results of the analyses on absolute responses. Indeed, the main effect of the target number was not significant, *F*(2, 72) = .330, *p* = .720, η^2^ = 1.754e^-4^, *VS-MPR* = 1.000, meaning that participants tended to consistently give the same response irrespective of the number of notes. In particular, nonmusicians tended to give answers close to 14 (*M =* 13.931, *SD* = 2.278), whereas musicians tended to give answers close to 15 (*M* = 15.369, *SD* = 1.413). The latter result is further highlighted by a significant main effect for the factor group in participants’ absolute responses, *F*(1, 36) = 5.782, *p* = .021, η^2^ = .127, *VS-MPR* = 4.463. Orthogonal contrasts showed that nonmusicians provided significantly lower absolute responses in all of the target numbers compared with musicians—14 notes: *t*(37.750) = −2.565, *p* = .014; 16 notes: *t*(37.750) = −2.282, *p* = .028; 18 notes: *t*(37.750) = −2.281, *p* = .028. More information about the mean absolute responses and mean error rates of musicians and nonmusicians in the control trials can be found in Table 3.

**Table 3:** Mean absolute responses and mean error rates (SD) of non-musicians and musicians in the control trials

	14 notes	16 notes	18 notes
Mean absolute responses			
Non-musicians	13.874 (2.210)	14.007 (2.306)	13.910 (2.358)
Musicians	15.414 (1.284)	15.377 (1.484)	15.315 (1.485)
Mean error rates			
Non-musicians	−0.893 (15.789)	−12.443 (14.422)	−22.7198 (13.103)
Musicians	10.099 (9.176)	−4.0108 (9.338)	−14.915 (8.247)

#### Comparison between test trials and control trials with the same number of piano notes

**Fig. 2:**
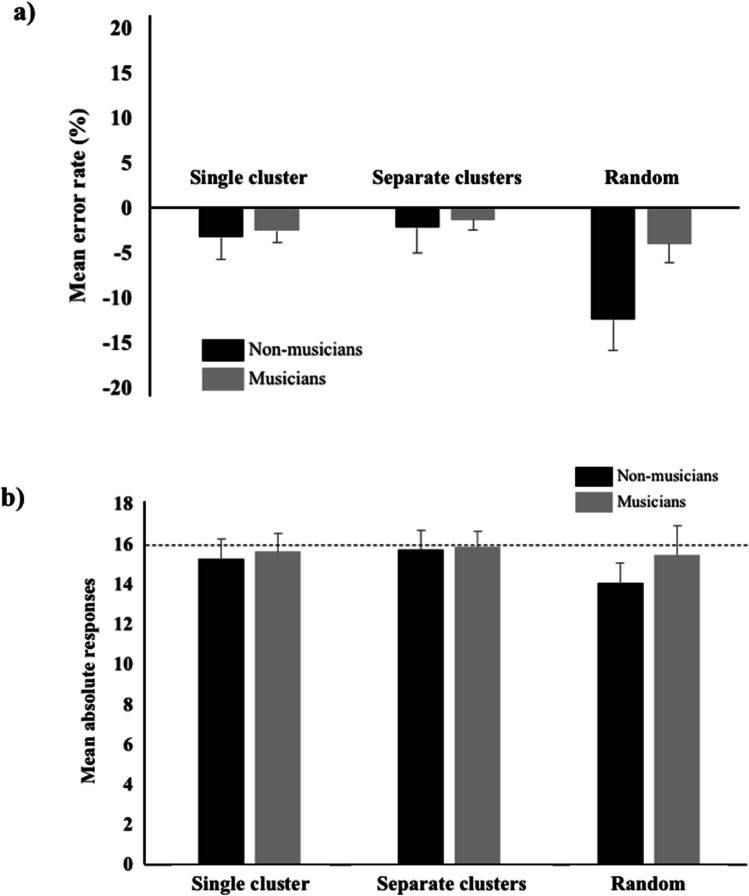
Results of test phase. (**a)** Mean error rates and (**b**) mean absolute responses of musicians and nonmusicians plotted against arrangements of the stimuli (piano notes ordered in a single cluster, ordered in two smaller clusters or randomly distributed). On average, nonmusicians showed a greater underestimation of 16 piano notes when they were randomly distributed. Means and standard errors are provided

Data analyses showed a significant main effect of the Arrangement, *F*(2, 72) = 12.121, *p* < .001, η^2^ = .062, *VS-MPR* = 1212.614, and a significant interaction between the group and arrangement factors, *F*(2, 72) = 4.460, *p* = .015, η^2^ = .023, *VS-MPR* = 5.862 (Fig. 2a). Contrast analysis showed that, whereas nonmusicians and musicians’ performances significantly differed in the case of 16 randomly located notes, *t*(59.542) = 2.683, *p* = .009, they did not differ either when piano notes were located to form a single cluster, *t*(59.542) = .774, *p* = .442, or when they were presented in two smaller separate clusters, *t*(59.542) = .275, *p* = .784. The analyses on the absolute responses showed that, in the illusory patterns, nonmusicians gave answers in line with those of musicians—single cluster: *t*(60.452) = .786, *p* = .435; two clusters: *t*(60.452) = .287, *p* = .775 (Fig. 2b). Indeed, all participants gave answers close to 16 (Table 4). Instead, when notes were randomly located, the absolute responses significantly differed as a function of the group, *t*(60.452) = 2.808, *p* = .007: The mean absolute response was 14.007 for nonmusicians and 15.377 for musicians. Moreover, nonmusicians gave different responses when notes where randomly located as compared with when they were regularly arranged (either in one or two clusters), *t*(72.000) = 5.346, *p* < .001. On the contrary, musicians’ absolute responses did not differ for notes arranged regularly and randomly, *t*(72.000) = 1.222, *p* = .226. More information about the mean absolute responses and mean error rates of musicians and nonmusicians in the illusory trials can be found in Table 4.

**Table 4:** Mean absolute responses and mean error rates (*SD*) of nonmusicians and musicians in the illusory trials

	Single cluster	Separate clusters	Random
Mean absolute responses			
Nonmusicians	15.199 (1.981)	15.645 (1.902)	14.007 (2.306)
Musicians	15.582 (0.905)	15.785 (0.818)	15.377 (1.484)
Mean error rates			
Nonmusicians	−3.261 (10.344)	−2.218 (11.891)	−12.443 (14.422)
Musicians	−2.604 (5.649)	−1.354 (5.115)	−4.011 (9.338)

Finally, as demonstrated in Tables 3 and 4, nonmusicians tended to show a higher variability in their responses compared with musicians.

#### Control test: Digit span test

No difference was found between musicians and nonmusicians, both when considering the overall number of items remembered, SF: *t*(38) = 0.923, *p* = 0.362; SB: *t*(38) = 0.229, *p* = 0.314, and when taking into account only the performance in the hardest trials, SSF: *t*(38) = 0.458,* p* = 0.650; SSB:* t*(38) = 1.269, *p* = 0.212.

## Discussion

Our goal was to test whether the differences observed between musicians and nonmusicians, in numerosity illusions based on Gestalt perception and regular/random displacement of the stimuli, could also be traced using the auditory modality. This would imply that the perceptual modifications associated with long-term music training are universal rather than modality dependent. To achieve this goal, we compared musicians and nonmusicians’ numerical estimation in the presence of an acoustic version of the Solitaire illusion (test trials) and stimuli in which nonordered sequences of notes were presented (control trials). We did not find evidence of differences in participants’ estimation when piano notes formed a single cluster rather than two smaller clusters. None of the different patterns (A, B, and C) played a significant role in participants’ responses. It is worth noting that, although our stimuli were created following the instructions provided by Prpic and Luccio (2016), they were also different with respect to two important issues. First, we opted for a tempo of 200 bpm (rather than 120 bpm) to avoid the possibility that musicians would have had more time to adopt metric strategies to estimate the number of piano notes. Second, we proposed a different task from that of Prpic and Luccio (2016), in which participants had to establish whether there were more piano or drums notes (i.e., a relative numerosity judgment). Instead, in our study, participants were presented with an absolute numerosity task in which they needed to estimate the number of piano notes. The lack of differences reported in our study as a function of the arrangements of the notes suggests that the emergence of an acoustic Solitaire illusion is not exclusively based on the pitch arrangements of the two types of sounds (piano and trombone notes) but may depend also on the physical properties of the stimuli (slower vs. faster tempo) and the task requests (relative vs. absolute numerosity judgments, as previously found with another visual illusion; Parrish et al., 2015). With respect to the comparison between the acoustic and visual arrangements of the items composing the illusory pattern, it is important to acknowledge that Pecunioso and Agrillo (2021) presented the most classical version of the illusion, the cross-like pattern, which is a spatial configuration that unfortunately cannot be transposed in the acoustic modality in any way. Here, we needed to adopt the linear versions of this illusion (Frith & Frith, 1972). We cannot exclude that the differential performance of musicians with the acoustic version of the Solitaire pattern might be due to slight differences in the magnitude of the illusory effect with the cross-like pattern and the linear version of the illusion.

As a secondary goal, we aimed to test whether regular patterns, in comparison with random ones, are also overestimated in the auditory modality (e.g., RRNI; Beran, 2006; Ginsburg, 1980). We found that clustering (ordered vs. random notes) does influence nonmusicians’ numerosity estimation. Indeed, nonmusicians were less accurate when piano notes were arranged regularly as compared with when they were randomly located. However, we noticed an important difference between the visual and auditory modalities: In the auditory modality, regularly arranged patterns were underestimated by nonmusicians, whereas the opposite effect was commonly reported in the visual modality (Beran, 2006; Ginsburg, 1980; Pecunioso et al., 2020). Hence, clustering might have a significant, but opposite, impact on the visual and auditory numerical estimation. One may hypothesize that relatively fast stimuli—like the ones used here—could increase the difficulty in segregating each sound. However, we believe that this is unlikely as the stimuli presented are below the threshold of gap detection used to delineate temporal resolution in the auditory system (Trehub et al., 1995). A different consideration must be made for musicians, where the “regular vs. random” bias was not observed. Musicians were similarly accurate for both regularly and randomly arranged patterns (with responses often close to 16). This is in line with Pecunioso and Agrillo’s (2021) study on the association between long-term musical training and a reduced susceptibility to numerosity illusions.

Analyses of the control trials highlighted a general tendency to underestimate target notes. Nonmusicians were more inclined to underestimate the number of notes compared with musicians. This is not unexpected in the literature as, in the visual modality, there is a general tendency to underestimate large numbers of items (Crollen et al., 2011; Izard & Dehaene, 2008; Krueger, 1982; Pecunioso et al., 2020; Zhang & Okamoto, 2017). Data reported here align with this literature and extend it to the auditory modality, suggesting that the tendency to underestimate large numbers is also modality independent. However, musicians estimated on average 15 notes in all control trials, whereas nonmusicians tended to perceive 14 notes. The mean absolute response, together with the lower variability of their responses, indicates that musicians were more accurate in the nonsymbolic numerical task. This issue is debated in the visual modality (Agrillo & Piffer, 2012; Pecunioso & Agrillo, 2021) and has never been reported, as far as we are aware, in the auditory modality. It is possible that the physiological enhancements throughout the auditory system associated with musical training (Rammsayer & Altenmuller, 2006; Strait & Kraus, 2014) lead to a better extrapolation of quantitative information from acoustic stimuli. Beyond this, the nature of the stimuli might have partially biased the result: to generate our stimuli, we used a software whose sounds are vaguely reminiscent of musical instruments. Also, in the familiarization phase we ensured that all participants could easily distinguish the two stimuli as a function of timbre. However, because musicians are more likely to be familiar with piano and trombone notes, we cannot exclude that a familiarity/perception advantage with these timbres might have occurred here.

One of the main difficulties in interpreting the differences between musicians and nonmusicians is that most of the studies are correlational ones that cannot firmly ascertain any far-transfer of skill from music training to other cognitive domains, as no direction of causality can be inferred. Also, because most of the studies investigating experts’ cognitive abilities are quasi-experiments, no random allocation of the participants can be done, a fact that prevents from safeguarding the baseline equivalence between the experimental and control groups. Recent meta-analyses have also argued against any possible association between musical training and enhanced cognitive skills (Sala & Gobet, 2017a, 2017b, 2020). We acknowledge that our study is another quasi-experiment with the same limits of existing literature (after all, our musicians differed for more than 10 years of musical practice). In determining the criteria for participants’ recruitment, we selected participants with a similar age, sex ratio‚ and education, limiting the intrinsic flaws of this widely adopted procedure. In addition, the fact that the digit span test did not show any difference between the two groups in both the control and test trials indicates that the phenomenon observed here cannot be ascribed to differences between the two groups in working memory (Talamini et al., 2017) and/or attentional/motivational factors.

To conclude, in agreement with what was observed in the visual modality, we found evidence that a specific clustering of acoustic stimuli (regular vs. ordered arrays) influences numerosity estimation, a fact that encourages the attempts to generate acoustic versions of the RRNI. This susceptibility seems different for individuals who received robust musical training, reinforcing the conclusions by Pecunioso and Agrillo (2021) on a link between musical training and different perceptual biases affecting numerosity estimation. However, the acoustic version of the Solitaire illusion presented here proved to be ineffective in generating a numerosity illusion based on the formation of a single Gestalt. The emergence of this illusion in the auditory modality is likely to be stimulus and task-dependent. Future investigations are now necessary to deepen the proper conditions in eliciting the Solitaire illusion in the auditory modality and to shed light on musicians’ susceptibility to acoustic numerosity illusions.

## Supplementary Information

Below is the link to the electronic supplementary material.

Supplementary file1 (DOCX 117 KB)

Supplementary file2 (ZIP 8.71 mb)
