# Supplementary material for: Investigating acoustic numerosity illusions in professional musicians
Source: Psychon Bull Rev. 2024 Apr 10;31(6):2611–20. doi: 10.3758/s13423-024-02496-2 (PMC11680625; doi:10.3758/s13423-024-02496-2)
Supplement: Supplementary file 1 — Supplementary file1 (DOCX 117 KB) [file 13423_2024_2496_MOESM1_ESM.docx]

| **PARTICIPANTS** | **GROUP** | **SEX** | **AGE** | **EDUCATION** |
| --- | --- | --- | --- | --- |
| 1 | Musician | F | 22 | Secondary School |
| 2 | Musician | F | 23 | Bachelor Degree |
| 3 | Musician | M | 26 | Bachelor Degree |
| 4 | Musician | F | 26 | Bachelor Degree |
| 5 | Musician | M | 21 | Secondary School |
| 6 | Musician | F | 24 | Bachelor Degree |
| 7 | Musician | M | 24 | Bachelor Degree |
| 8 | Musician | M | 22 | Secondary School |
| 9 | Musician | F | 22 | Secondary School |
| 10 | Musician | F | 26 | Bachelor Degree |
| 11 | Musician | F | 50 | Master Degree |
| 12 | Musician | M | 22 | Secondary School |
| 13 | Musician | M | 18 | Secondary School |
| 14 | Musician | F | 25 | Bachelor Degree |
| 15 | Musician | F | 28 | Master Degree |
| 16 | Musician | M | 23 | Bachelor Degree |
| 17 | Musician | F | 24 | Bachelor Degree |
| 18 | Musician | F | 28 | Master Degree |
| 19 | Musician | M | 25 | Bachelor Degree |
| 20 | Musician | F | 22 | Secondary School |
| 1 | Non-musician | M | 22 | Secondary School |
| 2 | Non-musician | F | 21 | Bachelor Degree |
| 3 | Non-musician | F | 22 | Secondary School |
| 4 | Non-musician | M | 22 | Secondary School |
| 5 | Non-musician | F | 24 | Bachelor Degree |
| 6 | Non-musician | M | 23 | Bachelor Degree |
| 7 | Non-musician | F | 25 | Master Degree |
| 8 | Non-musician | M | 24 | Bachelor Degree |
| 9 | Non-musician | F | 25 | Bachelor Degree |
| 10 | Non-musician | F | 22 | Secondary School |
| 11 | Non-musician | F | 21 | Secondary School |
| 12 | Non-musician | F | 24 | Bachelor Degree |
| 13 | Non-musician | M | 26 | Master Degree |
| 14 | Non-musician | M | 23 | Bachelor Degree |
| 15 | Non-musician | F | 23 | Bachelor Degree |
| 16 | Non-musician | M | 24 | Bachelor Degree |
| 17 | Non-musician | F | 24 | Bachelor Degree |
| 18 | Non-musician | F | 24 | Bachelor Degree |
| 19 | Non-musician | M | 26 | Master Degree |
| 20 | Non-musician | M | 23 | Bachelor Degree |
